# Cancer Patients Have an Increased Incidence of Dementia: A Retrospective Cohort Study of 185,736 Outpatients in Germany

**DOI:** 10.3390/cancers13092027

**Published:** 2021-04-22

**Authors:** Christoph Roderburg, Sven H. Loosen, Anselm Kunstein, Raphael Mohr, Markus S. Jördens, Mark Luedde, Karel Kostev, Tom Luedde

**Affiliations:** 1 Clinic for Gastroenterology, Hepatology and Infectious Diseases, University Hospital Düsseldorf, Medical Faculty of Heinrich Heine University Düsseldorf, Moorenstraße 5, 40225 Düsseldorf, Germany; christoph.roderburg@med.uni-duesseldorf.de (C.R.); sven.loosen@med.uni-duesseldorf.de (S.H.L.); anselm.kunstein@med.uni-duesseldorf.de (A.K.); markus.joerdens@med.uni-duesseldorf.de (M.S.J.); 2 Department of Hepatology and Gastroenterology, Charité University Medicine Berlin, Augustenburger Platz 1, 13353 Berlin, Germany; raphael.mohr@charite.de; 3 KGP Bremerhaven, 27580 Bremerhaven, Germany; mark.luedde@web.de; 4 Epidemiology, IQVIA, 60549 Frankfurt, Germany; Karel.Kostev@iqvia.com

**Keywords:** cognitive impairment, malignancy, malignant melanoma

## Abstract

**Simple Summary:**

Cancer is the second leading cause of death worldwide and incidence rates for several tumor entities are rising. Many patients develop additional comorbidities after cancer diagnosis. Among these, several psychological morbidities have been extensively studied in the past, but findings on the association between cancer and dementia have remained conflicting. We showed that the overall cumulative incidence of dementia was significantly higher in cancer patients than in non-cancer patients, which should raise awareness of this important comorbidity in cancer patients.

**Abstract:**

Background: Cancer is the second leading cause of death worldwide and incidence rates for several tumor entities are rising. In addition to a high cancer-specific mortality rate, many cancer patients also suffer from additional comorbidities. Among these, several psychological morbidities have been extensively studied in the past, but findings on the association between cancer and dementia have remained conflicting. In the present study, we evaluated the possibility of an association between cancer and dementia. Methods: Based on data from the IQVIA Disease Analyzer database, a total of 92,868 cancer outpatients initially diagnosed between 2000 and 2018 were matched by age, gender, index year, and yearly consultation frequency to 92,868 individuals without cancer. Ten-year incidence rates of dementia were compared for the two cohorts. Results: The overall cumulative incidence of dementia was significantly higher in cancer patients (19.7%) than in non-cancer patients (16.7%, *p* < 0.001). Cox regression models confirmed that this association was significant for both male (HR: 1.35 [1.30–1.41], *p* < 0.001) and female (HR: 1.26 [1.21–1.31], *p* < 0.001) patients and was consistent among all age groups analyzed (65–70, 71–75, 76–80, 81–85, and >85 years). In addition, the association between cancer and dementia was significant for all cancer entities analyzed (skin, digestive organs, prostate, breast, urinary tract, lymphoid and hematopoietic tissue, and lung cancer) and most pronounced in patients with lung cancer (HR: 1.44 [1.28–1.62], *p* < 0.001). Conclusions: Our data provide strong evidence for an increased incidence of dementia in a large cohort of patients with different cancer entities, which should raise awareness of this important comorbidity in cancer patients.

## 1. Introduction

With increasing life expectancy, both the incidence and prevalence of age-related diseases such as cancer and dementia have risen dramatically. Indeed, cancer is already the most common cause of death in many European countries [1]. At the same time, the prevalence of cognitive disease in older populations ranges from 4% to 30%, depending on which definition is applied [2]. In addition, over 50 million patients are diagnosed with dementia each year, a number which almost doubles every 20 years [3], highlighting the magnitude of the problem.

As cancer and dementia affect a similar collective of patients, numerous analyses in the past have addressed the possibility of a causal relationship between the two diseases and investigated whether cancer may be causal for dementia, but also whether dementia can lead to cancer (summarized, e.g., in [4]). Cognitive impairment in the form of what is referred to as “chemo brain” might indicate an association between the two diseases [5]. However, recent longitudinal studies on patients receiving chemotherapy for cancer have demonstrated that chemotherapy may not be the only cause of cognitive problems, as some found that patients already showed lower than expected cognitive function before the start of chemotherapy [6,7,8]. Furthermore, imaging studies demonstrated that cancer patients already displayed altered structural and functional brain structures before the initiation of systemic cancer treatment [9,10]. On a molecular basis, systemic inflammatory processes, which are characteristic for cancer and have also been found in dementia patients, may constitute a link between the two diseases [11]. In recent years, the association between cancer and cognitive impairment has been analyzed in multiple population-based studies. However, contrary to general expectations, patients with cancer in some of these analyses demonstrated a decreased risk of dementia (summarized, e.g., in [4]). Other analyses reported that these inverse correlations between cancer and dementia might be restricted to specific etiologies such as Alzheimer’s disease and diminish over time, arguing that the association between cancer and general risk of dementia is limited [12]. Furthermore, other authors reported a positive association between tumor markers and dementia rates in cancer patients [13].

In the context of these conflicting data, we evaluated the incidence of dementia in patients with various types of cancer and matched individuals without cancer.

## 2. Methods

### 2.1. Database

This study was based on data from the Disease Analyzer database (IQVIA), which contains drug prescriptions, diagnoses, and basic medical and demographic data obtained directly and in anonymous format from computer systems used in the practices of general practitioners and specialists [14]. The database covers approximately 3% of all outpatient practices in Germany. Diagnoses (according to the International Classification of Diseases, 10th revision (ICD-10)), prescriptions (according to the Anatomical Therapeutic Chemical (ATC) classification system), and the quality of reported data are monitored on a regular basis by IQVIA. In Germany, the sampling methods used to select physicians’ practices are appropriate for obtaining a representative database of general and specialized practices. It has previously been shown that the panel of practices included in the Disease Analyzer database is representative of general and specialized practices in Germany [14]. For example, Rathmann et al. demonstrated a good agreement between the outpatient DA database and the German reference data in terms of the incidence or prevalence of cancer diagnoses [14]. Finally, this database has already been used in previous studies focusing on dementia [15] as well as cancer [16].

### 2.2. Study Population

**Figure 1:**
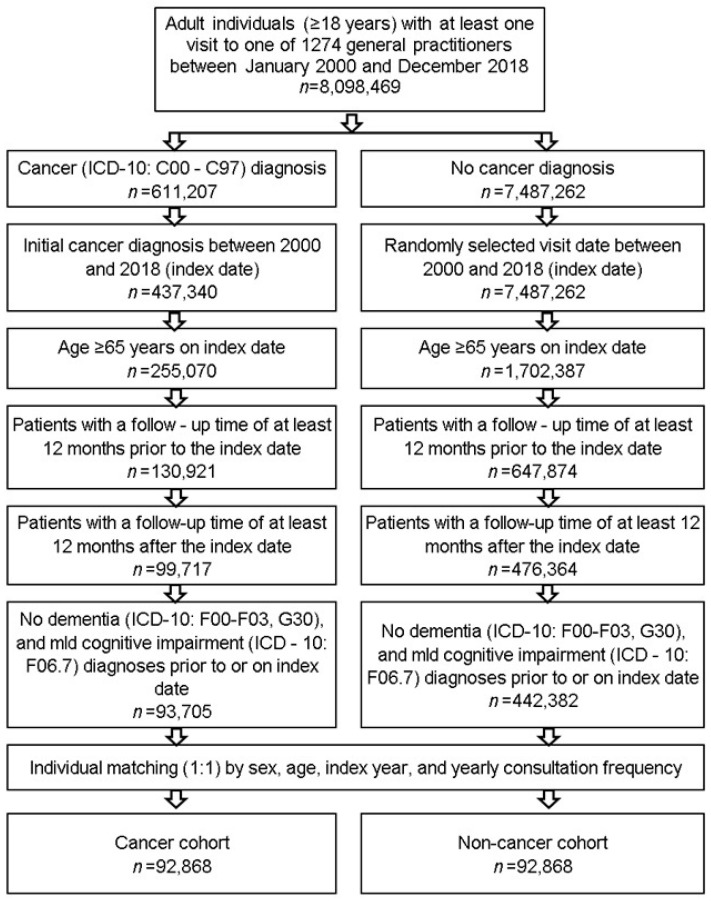
Selection of study patients.

This retrospective cohort study included patients ≥ 65 years with an initial diagnosis of cancer (ICD-10: C00-C97) in 1274 general practices in Germany between January 2000 and December 2018 (index date; Figure 1). One further inclusion criterion was an observation time of at least 12 months prior to the index date and at least 12 months after the index date. In addition, patients with dementia (ICD-10: F00-F03, G30) or mild cognitive impairment (ICD-10: F06.7) diagnoses prior to the index date were excluded to allow the incident diagnosis of dementia to be estimated after the index date. Cancer patients were matched to non-cancer patients by sex, age, index year, and yearly consultation frequency. For the non-cancer patients, the index date was that of a randomly selected visit between January 2000 and December 2018.

### 2.3. Study Outcomes and Covariates

The main outcome of the study was the incidence of dementia among patients with cancer compared with non-cancer patients. As more than 80% of dementia patients have unspecified dementia (ICD-10: F03), all dementia types were analyzed as a compound effect.

### 2.4. Statistical Analyses

Differences in the sample characteristics between patients with or without cancer were investigated using chi-squared tests for categorical variables and Wilcoxon tests for continuous variables. In addition to age and sex, the cohorts were compared in terms of several comorbidities documented within 12 months prior to the index date: Diabetes mellitus (ICD-10: E10-14), obesity (ICD-10: E66), hypertension (ICD-10: I10), lipid metabolism disorders (ICD-10: E78), peripheral artery disease (ICD-10: I70.2, I73.9), myocardial infarction (ICD-10: I21-I23, I25.2), and stroke, including TIA (ICD-10: I63, I64, G45), depression (ICD-10: F32, F33), and osteoporosis (ICD-10: M80, M81). The cumulative incidence of dementia within ten years after the index date was evaluated using Kaplan–Meier curves. Multivariable Cox regression models were used to study the association between cancer and dementia, adjusted for co-morbidities. These models were applied separately for five age groups (65–70, 71–75, 76–80, 81–85, >85 years) as well as for women and men. Finally, these models were used for each of the most common cancer sites (including breast (ICD-10: C50), prostate (ICD10: C61), skin (ICD-10: C43, C44), lung (ICD-10: C34), digestive organs (ICD-10: C15-C26), urinary tract (ICD-10: C64-C68), and lymphoid and hematopoietic tissue (ICD-10: C81-C96)) versus matched non-cancer patients. As 15 models were used, a Bonferroni correction for *p*-value was performed, and a *p*-value of <0.003 (calculated as <0.05/15) was considered statistically significant. Analyses were carried out using SAS version 9.4 (SAS Institute, Cary, NC, USA).

## 3. Results

### 3.1. Basic Characteristics of Study Cohort

To investigate a potential association between cancer and dementia, we identified a total of 92,868 cancer patients who were matched to a cohort of equal size without cancer. The majority of patients had a diagnosis of skin cancer (*n* = 21,716), followed by cancer of the digestive organs (*n* = 14,340), prostate cancer (*n* = 14,001), breast cancer (*n* = 11,708, females only), urinary tract cancer (*n* = 7.290), lymphoid and hematopoietic tissue cancer (*n* = 6369), and lung cancer (*n* = 3500). The mean age of all cancer patients was 74.8 years (SD: 6.4 years). Of the patients, 52.1% were male and 47.9% were female. Table 1 summarizes the basic characteristics of the study cohort.

### 3.2. Incidence Rates of Dementia Are Higher in Cancer Patients

**Figure 2:**
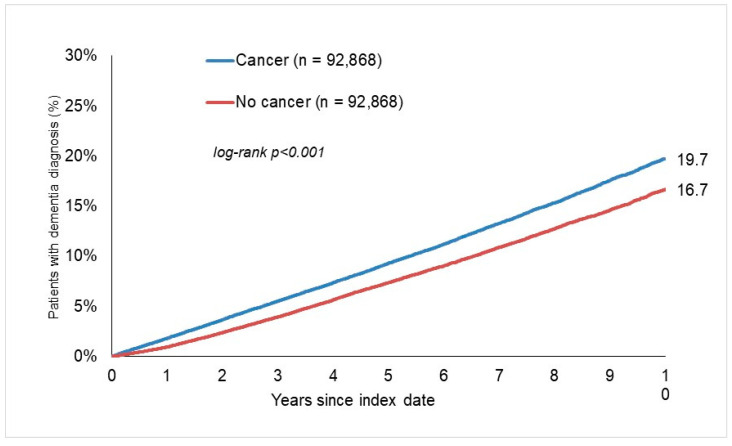
Ten-year cumulative incidence of dementia diagnoses in patients with and without cancer.

We first compared the overall incidence of dementia within a 10-year observation period in cancer patients with that in matched patients without cancer (Figure 1). Interestingly, the 10-year-cumulative incidence of dementia was significantly higher in cancer patients (19.7%) than in non-cancer patients (16.7%, *p* < 0.001). This finding was confirmed in a Cox regression model showing a hazard ratio of 1.30 (95%-CI: 1.27–1.34) for the incidence of dementia in cancer patients (Figure 2).

### 3.3. Subgroup Analysis of Demographic Aspects and Tumor Entities

Subsequently, we evaluated whether the significant association between cancer and dementia was restricted to certain demographic subgroups or only observed in the presence of particular cancer entities. We observed a significant association between cancer diagnoses and the incidence of dementia in both male (HR: 1.35 [1.30–1.41], *p* < 0.001) and female (HR: 1.26 [1.21–1.31], *p* < 0.001) patients (Table 2). In addition, this association was consistent among all age groups analyzed (65–70, 71–75, 76–80, 81–85, and >85 years, Table 2). When comparing different tumor entities, we found a significant association between cancer and dementia in all analyzed subgroups (skin, digestive organs, prostate, breast, urinary tract, lymphoid and hematopoietic tissue, and lung cancer, Table 2). Interestingly, the association was strongest in patients with lung cancer (HR: 1.44 [1.28–1.62], *p* < 0.001).

## 4. Discussion

The specific association between cancer and the brain has been analyzed intensively with at least partially conflicting results [17,18,19,20]. While many cancer patients complain of intermittent impaired cognitive performance, the association between cancer and the development of dementia in the long-term follow-up period remains highly controversial. In this study, we provided strong evidence for an increased incidence of dementia in a large cohort of patients with different cancer entities. We showed that the incidence of dementia is significantly higher in cancer patients (19.7%) than in non-cancer patients (16.7%, *p* < 0.001). Cox regression models confirmed that this association was significant in both male and female patients and was consistent among all age groups analyzed. In addition, the association between cancer and dementia was significant among all cancer entities analyzed but most pronounced in patients with lung cancer.

In contrast to our study, recent population-based analyses have suggested an inverse link between cancer and the development of dementia, particularly Alzheimer’s disease [21,22,23]. In addition to the direct effects of cancer, various retrospective studies have analyzed the effects of chemotherapy on dementia with at least partially conflicting results [24,25,26,27]. The investigation of the association between cancer and dementia is complicated by a number of methodological issues. Most importantly, survival bias and surveillance bias impede the interpretation of many of the studies available and might be responsible for inter-study differences. Considering the high rates of cognitive problems in patients suffering from cancer as well as shared genetic traits, many authors have questioned whether there actually is a “true” reduction in the risk of dementia in cancer patients [4]. A positive association seems more probable from a clinical and pathophysiological perspective. Notably, similar molecular mechanisms including inflammation, oxidative stress, and angiogenesis have been linked with both cancer and dementia. Both animal models and clinical data from humans have demonstrated impaired cognitive capability and a deterioration of mental abilities in the context of inflammation, reflected by elevated serum concentrations of fibrinogen and interleukin-6. In addition, accumulation of the amyloid beta (Aβ) peptide has been described both in patients with Alzheimer’s disease and in those with several types of tumors, and was associated with cell proliferation, migration, and invasion [28]. Elevated plasma amyloid-beta levels have also been found in patients with different types of cancer [29]. In line with these findings, BRCA1, an important tumor suppressor protein, has recently been linked to Alzheimer’s disease and is thought to promote neuronal cell death [30]. Finally, various mechanisms leading to DNA damage (e.g., oxidative stress, deficient DNA repair mechanisms) have been recognized as significant features of cancer and dementia, potentially connecting both diseases. Interestingly, syndromes associated with genetic defects in DNA damage repair mechanisms (e.g., xeroderma pigmentosum) are characterized by an increased risk of cancer and cognitive problems, supporting the hypothesis of a common pathophysiology of both diseases [31].

Neurotoxicity from cancer treatment represents a frequent event in systemic treatment of malignancies. Given the protection of the blood–brain, blood–cerebrospinal fluid, and blood–nerve barriers, as well as the low reproductive rate of neurons, the nervous system should be relatively protected from chemotherapy and radiotherapy toxicities [31]. However, neurotoxicity is the second most common cause dose-limiting factor of cancer treatment [32]. Neurotoxicity can manifest in many different ways and treatments affect both the central (headache, seizures, encephalopathy, movement disorders, cerebellar dysfunction, and ataxia) and peripheral (plexopathy, peripheral neuropathy, and inflammatory demyelinating polyneuropathy) nervous systems, supporting the hypothesis that systemic chemotherapy might cause dementia. Mechanisms leading to neurotoxicity are complex and highly dependent on the used substance, the dosage route of administration, and pre-existing disease conditions. Classical cytotoxic substances causing neurotoxicity are methotrexate, vinca alkaloids, and platinums, to name a few [32]. Nevertheless, newer therapeutic agents including brentuximab vedotin and blinatumomab might also cause sever neurotoxicity in cancer patients [32]. Finally, immune check-point inhibitors have been associated with neurotoxicities including meningoencephalitis, myasthenia gravis, and various neuropathies [33]. Together with our data, these findings suggest that the different forms of acute and chronic neurotoxicity are a significant clinical problem in all patients receiving systematic chemotherapy, and patients must be monitored and treated accordingly.

While our study was not designed to provide any molecular insights into mechanisms promoting (or preventing) dementia in cancer patients, our data should raise awareness of this important comorbidity in cancer patients. In line with our data, it was recently demonstrated that elevated levels of carcinoembryonic antigen might be predictive for the development of dementia in cancer patients [13]. Such associations highlight the need for further research, potentially using preclinical cancer and dementia models, in order to establish better prevention and therapeutic strategies for both diseases. Such considerations are particularly important in view of the fact that incidence rates of cancer are rising globally, especially in developed countries with relatively old populations. Due to recent advances in cancer treatment strategies, many cancers represent chronic diseases. Therefore, the question of sequelae, which can often only be observed in the long term, has become a significant issue in the clinical management of cancer patients.

In conclusion, our data from a large German cohort of cancer-patients suggest a positive association between cancer and the development of dementia. Similarly to previous studies (see above), our study was subject to several limitations that were due to the study design and either could not be avoided at all or could not effectively be avoided in studies based on database analyses, as recently described [34]. First, the ICD-10 coding system was used, which might have led to misclassification and undercoding of certain diagnoses. Second, general practitioners in Germany document only three dementia-related ICD-10 codes, namely vascular dementia (ICD-10: F01), Alzheimer’s disease (ICD-10: G30), and other and unspecified dementia (ICD-10: G03). In our analysis of patients with dementia, Alzheimer’s disease was documented in 11.8% of cancer and 11.5% of non-cancer patients, vascular dementia in 17.6% of cancer and 16.4% of non-cancer patients, and other and unspecified dementia in 70.6% of cancer and 72.1% of non-cancer patients. Third, the German Disease Analyzer database does not record larger panels of laboratory values or histological parameters, meaning that it was not possible to conduct analyses correlating tumor stage with effect size. Furthermore, we were unable to analyze associations with possible neurodegenerative effects of anticancer pharmacological treatments since the individual medications are not included in the database. In addition, we could not draw conclusions about potential associations with psychological distress. Similarly, data on socioeconomic status (e.g., education and income) and lifestyle-related risk factors (e.g., smoking, alcohol consumption, and physical activity) were also lacking. Additionally, the German Disease Analyzer database is dedicated to the analysis of data from outpatients and features no data from patients treated in hospital settings. It is also not possible to display time courses, meaning that we could not state whether a successful treatment of cancer might have been associated with a decreased incidence of dementia. Finally, we could not exclude the possibility of selection bias in our study for those with cancer, meaning that patients who have an established diagnosis of cancer may be more likely to be examined for dementia. Nevertheless, our study is the first to demonstrate an association between cancer and dementia in a German cohort and might provide further answers on the important question of the association between both diseases.

## 5. Conclusions

Our study is the first to demonstrate an association between cancer and dementia in a German cohort and might provide further answers on the important question of the association between both diseases.

## Figures and Tables

**Table 1:** Basic characteristics of the study sample after 1:1 matching.

Variable	Proportion Affected among Patients with Cancer (%) *n* = 92,868	Proportion Affected among Patients without Cancer (%) *n* = 92,868	*p*-Value
**Age (mean, SD)**	74.8 (6.4)	74.8 (6.4)	1.000
**Age 65–70**	29.6	29.6	1.000
**Age 71–75**	27.2	27.2	
**Age 76–80**	23.6	23.6	
**Age 81–85**	13.3	13.3	
**Age >85**	6.3	6.3	
**Women**	47.9	47.9	1.000
**Men**	52.1	52.1	
**Number of visits per year during the follow-up time**	6.8 (6.2)	6.8 (6.2)	1.000
**Comorbidities documented within 12 months prior to index date**			
**Diabetes**	30.2	34.1	<0.001
**Obesity**	11.6	12.3	<0.001
**Hypertension**	69.7	72.0	<0.001
**Lipid metabolism disorders**	46.7	47.6	<0.001
**Peripheral artery disease**	8.0	8.2	0.039
**Myocardial infarction**	3.5	3.6	0.033
**Stroke including TIA**	7.4	8.0	<0.001
**Depression**	17.2	17.8	0.004
**Osteoporosis**	11.7	11.3	0.009

Proportions of patients are indicated in % unless otherwise indicated. SD: Standard deviation.

**Table 2:** Association between cancer and incident dementia within 10 years of the index date in patients followed in general practices in Germany (Cox regression models).

Variable	Hazard Ratio (95% CI) *	*p*-Value
**Total (*n* = 92,868 pairs)**	1.30 (1.27–1.34)	<0.001
**Age 65–70 (*n* = 27,484 pairs)**	1.33 (1.24–1.43)	<0.001
**Age 71–75 (*n* = 25,221 pairs)**	1.31 (1.24–1.39)	<0.001
**Age 76–80 (*n* = 21,958 pairs)**	1.26 (1.20–1.33)	<0.001
**Age 81–85 (*n* = 12,326 pairs)**	1.25 (1.17–1.33)	<0.001
**Age >85 (*n* = 5879 pairs)**	1.30 (1.19–1.42)	<0.001
**Female patients (*n* = 44,461 pairs)**	1.26 (1.21–1.31)	<0.001
**Male patients (*n* = 48,407 pairs)**	1.35 (1.30–1.41)	<0.001
**Breast cancer (women) (*n* = 11,708 pairs)**	1.23 (1.15–1.31)	<0.001
**Prostate cancer (men) (*n* = 14,001 pairs)**	1.27 (1.20–1.35)	<0.001
**Skin cancer (*n* = 21,716 pairs)**	1,29 (1.24–1.35)	<0.001
**Lung cancer (*n* = 3500 pairs)**	1.44 (1.28–1.62)	<0.001
**Digestive organ cancer (*n* = 14,340 pairs)**	1.28 (1.22–1.35)	<0.001
**Lymphoid and haematopoietic tissue cancer (*n* = 6369 pairs)**	1.24 (1.14–1.34)	<0.001
**Urinary tract cancer (*n* = 7290 pairs)**	1.24 81.15–1.33)	<0.001

* Multivariable Cox regression adjusted for co-morbidities (diabetes, obesity, hypertension, lipid metabolism disorders, peripheral artery disease, myocardial infarction, and stroke, including TIA (transient ischemic attack), depression, and osteoporosis).

## Data Availability

Data are available upon meaningful request from the corresponding author.
